# Usefulness of fibrosis-4 (FIB-4) score and metabolic alterations in the prediction of SARS-CoV-2 severity

**DOI:** 10.1007/s11739-022-03000-1

**Published:** 2022-06-26

**Authors:** Rosa Lombardi, Vincenzo La Mura, Annalisa Cespiati, Federica Iuculano, Giordano Sigon, Giada Pallini, Marco Proietti, Irene Motta, Beatrice Montinaro, Elisa Fiorelli, Matteo Cesari, Alessandra Bandera, Luca Valenti, Flora Peyvandi, Nicola Montano, Marina Baldini, Anna Ludovica Fracanzani

**Affiliations:** 1 Unit of Internal Medicine and Metabolic Diseases, Fondazione IRCCS Ca’ Granda Ospedale Maggiore Policlinico, Milan, Italy; 2 Department of Pathophysiology and Transplantation, Università Degli Studi di Milano, Via F. SFORZA 35, 20122 Milan, Italy; 3 Unit of Internal Medicine Hemostasis and Thrombosis, Fondazione IRCCS Ca’ Granda Ospedale Maggiore Policlinico, Milan, Italy; 4 Department of Biomedical Sciences for Health, Università degli Studi di Milano, Milan, Italy; 5 Geriatric Unit, IRCCS Istituti Clinici Scientifici Maugeri, Milan, Italy; 6 Department of Clinical Sciences and Community Health, Università degli Studi di Milano, Milan, Italy; 7 Unit of Internal Medicine, Fondazione IRCCS Ca’ Granda Ospedale Maggiore Policlinico, Milan, Italy; 8 Unit of Internal Medicine and Immunology, Fondazione IRCCS Ca’ Granda Ospedale Maggiore Policlinico, Milan, Italy; 9 Unit of Infectious Diseases, Fondazione IRCCS Ca’ Granda Ospedale Maggiore Policlinico, Milan, Italy; 10 Department of Transfusion Medicine and Hematology, Università degli Studi di Milano, Milan, Italy

**Keywords:** Type 2 diabetes, Metabolic burden, FIB-4, Hepatic steatosis, Respiratory failure

## Abstract

Despite vaccination programs, severe acute respiratory syndrome coronavirus 2 (SARS-CoV-2) infection remains a public health problem. Identifying key prognostic determinants of severity of the disease may help better focus health resources. The negative prognostic role for metabolic and hepatic alterations is established; however, the interplay among different metabolic comorbidities and their interconnections with the liver have never been explored.

The objective of this study is to evaluate the impact of liver alterations in addition to metabolic comorbidities as a predictor of SARS-CoV-2 severity. 382 SARS-CoV-2 patients were enrolled. Severe SARS-CoV-2 was diagnosed according to international consensus. Transaminases > 2 times the upper limit of normality (2ULN), hepatic steatosis (by ultrasound and/or computed tomography in 133 patients), and FIB-4 defined liver alterations. All data were collected on admission. The results are severe SARS-CoV-2 infection in 156 (41%) patients (mean age 65 ± 17; 60%males). Prevalence of obesity was 25%; diabetes, 17%; hypertension, 44%; dyslipidaemia, 29%; with 13% of the cohort with ≥ 3 metabolic alterations. Seventy patients (18%) had transaminases > 2ULN, 82 (62%) steatosis; 199 (54%) had FIB-4 < 1.45 and 45 (12%) > 3.25. At multivariable analysis, ≥ 3 metabolic comorbidities (OR 4.1, CI 95% 1.8–9.1) and transaminases > 2ULN (OR 2.6, CI 95% 1.3–6.7) were independently associated with severe SARS-CoV-2. FIB-4 < 1.45 was a protective factor (OR 0.42, CI 95% 0.23–0.76). Hepatic steatosis had no impact on disease course. The presence of metabolic alterations is associated with severe SARS-CoV-2 infection, and the higher the number of coexisting comorbidities, the higher the risk of severe disease. Normal FIB-4 values are inversely associated with advanced SARS-CoV-2 regardless of metabolic comorbidities, speculating on use of these values to stratify the risk of severe infection.

**Supplementary Information:**

The online version contains supplementary material available at 10.1007/s11739-022-03000-1.

## Introduction

COVID-19 is an acute disease sustained by severe acute respiratory syndrome coronavirus 2 (SARS-CoV-2) infection, possibly leading to respiratory failure and death. Along with respiratory symptoms, a rise in liver enzymes has been reported, especially in severe COVID-19 [1].

Despite vaccination programs, COVID-19 infection remains today one of the main public health problems in patients at risk. Indeed, identifying the key prognostic determinants of severity of the disease may help better focus economic and health resources.

Metabolic comorbidities emerged as key prognostic determinants of aggressive viral disease. Indeed, type 2 diabetes (T2DM) has been linked to a greater risk of critical illness and in-hospital mortality [2, 3]. In addition, arterial hypertension has been demonstrated to predispose to worse respiratory exchanges in patients admitted to intensive-care units (ICU) [4]. Finally, obesity has been associated with a higher rate of ventilation support, hospitalization, and morbidity from SARS-CoV-2 [5–7]. More recently, non-alcoholic fatty liver disease (NAFLD) and advanced fibrosis assessed by FIB-4 have been possibly associated with a higher prevalence of severely ill COVID-19 infection; however, data are contrasting and not conclusive [8–10].

Data on metabolic alterations and COVID-19 emerged from observational studies mainly in Asian populations, often including patients admitted to ICU [2, 11].

In addition, the effect of the single or differently combined features of metabolic syndrome (e.g., obesity, dyslipidaemia, diabetes, and arterial hypertension) on COVID-19 presentation and outcome has never been explored, nor the hepatic alterations have been evaluated as an additional risk factor for the severity of the respiratory disease.

The aim of our study was to define a possible prognostic factor of the severity of COVID-19 infection. The first aim was to evaluate how different metabolic comorbidities, considered either singularly or coexisting, foster the severity of respiratory COVID-19 infection and mortality in a cohort of Italian patients admitted to non-intensive hospital units.

As a secondary aim, we explored the impact of liver alterations in addition to metabolic comorbidities on COVID-19 outcomes in the same cohort of patients.

## Patients and methods

### Patients

In this retrospective observational study, we enrolled 382 patients with confirmed SARS-CoV-2 infection and aged > 18 years who referred to three non-intensive units between 01 March and 01 May 2020. Patients were transferred from the Emergency Department, Sub-intensive and Intensive care Units (ICU) of Policlinico Hospital of Milan or from other hospitals in the Lombardy region.

The diagnosis of SARS-CoV-2 was confirmed by real-time reverse-transcription polymerase chain reaction (RT-PCR) analysis.

Patients with active congestive heart failure, known liver disease, type 1 diabetes mellitus, pregnancy, hematologic disease, and life expectancy of less than 6 months were excluded.

The study protocol was approved by the Institutional Review Board. For all patients, informed consent to participate the study was obtained, according to the ethical guidelines of the 1975 Declaration of Helsinki.

### Assessment of metabolic comorbidities

At admission, clinical and anthropometric data were collected and a biochemical panel, including glucose and lipid profiles, was performed for all patients.

Body mass index (BMI) < 25, 25–29, and ≥ 30 kg/m^2^ defined the presence of normal weight, overweight, and obesity in 271 patients with available data. Hypertension, dyslipidaemia, and type 2 diabetes (T2DM) were defined according to the international consensus [12–14] or retrieved by medical history.

### Assessment of liver disease

At admission, liver enzymes were considered increased for aspartate aminotransferase (AST) values ≥ 39 U/L and alanine aminotransferase (ALT) ≥ 41 U/L, according to local laboratory cut-offs. Values 2 times the upper limit of normality (2ULN) defined significant hypertransaminasemia.

Presence of hepatic steatosis was diagnosed by abdomen ultrasound (US) [15] and/or chest or abdomen computed tomography (CT) scan [16] performed in 133 patients during hospitalization for clinical reasons related to COVID disease and retrospectively analyzed.

To further evaluate liver alterations, we applied the Fibrosis-4 (FIB-4), a non-invasive biomarker used to exclude or detect the presence of hepatic fibrosis by a dual cut-off, 1.45 and 3.25, and calculated by the formula [17].

$$ FIB4 = \frac{{{\text{Age}}\left( {{\text{years}}} \right) \times AST\left( \frac{U}{L} \right)}}{{{\text{Platelets}}\left( {10^{9} } \right) \times \sqrt {ALT} \left( \frac{U}{L} \right)}}. $$

To calculate this score, patients with thrombocytopenia or thrombocytosis due to hematologic disease were not considered, as well as patients with transaminases > 5ULN to rule out an acute hepatitis [18].

To exclude a possible influence of alcohol consumption on liver disease, patients with alcohol intake of more than 20 g/day (women) or 30 g/day (men) were not included in the analysis, according to International Guidelines [19]. Information about alcohol intake was retrieved by medical history or by medical records in patients with compromised clinical conditions.

### Assessment of severity of COVID-19

Severity of COVID-19 disease was defined according to the American Thoracic Society guidelines for community-acquired pneumonia [20]. In particular, we focused on specific severe respiratory manifestations of COVID-19 infection at admission or during hospitalization as the onset of a respiratory failure requiring mechanical ventilation or non-invasive ventilation, such as continuous positive airway pressure (CPAP), and/or a respiratory rate (RR) ≥ 30 breaths/minutes and/or a PaO2/FiO2 (P/F) ratio ≤ 200 at arterial blood gas test at presentation, as described in Supplementary Table 1.

Given the evidence of a rise in ferritin, D-dimer, and Inteleukin-6 (IL-6) [21] during severe COVID-19 disease, an inflammatory panel including these biomarkers was performed at admission.

In addition, as per local guidelines of the Infectious Diseases Unit of our Hospital, patients were treated during hospitalization with steroidal therapy (24% of the cohort) and low-weight heparin (83%), and received at least one antiviral drug, among which hydroxychloroquine, remdesivir, and lopinavir/ritonavir (84%).

### Statistical analysis

Continuous variables were expressed as media and standard deviation (SD) and median and interquartile range (IQR) for normally and non-normally distributed variables, while categoric variables as absolute and relative frequencies (n,%). Continuous variables were compared using Student’s t test or Mann–Whitney test for normally and non-normally distributed variables, and ANOVA when appropriate. Differences between categorical variables were examined by the Chi-square test. Sample size was not calculated because of the retrospective observational design of the study.

Differences in prevalence of metabolic comorbidities, hepatic steatosis, and FIB4 values < or > 1.45 in patients with and without features of severe COVID-19 infection were evaluated. The association between metabolic comorbidities and severe COVID-19 infection and mortality was assessed by binary logistic regression corrected for hepatic disease and inflammatory markers, and a maximum of one variable for each 10 patients with severe COVID-19 infection was added to the model.

A two-tailed *p* value < 0.05 was considered statistically significant. Statistical analysis was carried out using SPSS package, version 26.

## Results

### Characterization of the cohort at admission

#### Demographic and metabolic characteristics

Mean age was 65 ± 17 years, 60% of patients were male, 85% Caucasians. Mean BMI was 27 ± 5 kg/m^2^, with 61% of patients overweight and 25% obese. Prevalence of hypertension was 44%, dyslipidaemia 29% (mean LDL 94 ± 45 mg/dL, mean HDL 41 ± 17 mg/dL, median triglycerides 127 mg/dL) and T2DM 17% (mean glucose 120 ± 47 mg/dL) (Table 1).

**Table 1:** Demographic, clinical, and biochemical characteristics of the whole cohort of SARS-CoV-2 infected patients (*n *= 382), stratified by SARS-CoV-2 infection severity

	Total (*n *= 382)		Non-severe SARS-CoV-2 infection (*n *= 226)		Severe SARS-CoV-2^a^ infection (*n *= 156)		*p *value
General characteristics							
Age, yrs	65 ± 17		65 ± 18		65 ± 15		0.5
Sex male, *n* (%)	229 (60)		127 (56)		102 (65)		0.09
Ethnicity, *n* (%)	Caucasic	325(85)	Caucasian	187(83)	Caucasian	138(88)	0.2
	Arabic	14(4)	Arabic	8(4)	Arabic	6(4)	
	African	2(1)	African	2(1)	African	0(0)	
	Hispanic	32(8)	Hispanic	21(9)	Hispanic	11(7)	
	Asian	9(2)	Asian	8(3)	Asian	1(1)	
Active smoking, *n* (%)	19 (5)		15 (6)		4 (3)		0.3
Metabolic comorbidities							
BMI^b^, kg/m.^2^	27 ± 5		27 ± 6		27 ± 5		0.8
> 25, *n* (%)	166 (61)		88 (59)		78 (64)		0.4
> 30, *n* (%)	69 (25)		34 (23)		35 (29)		0.3
Hypertension, *n* (%)	169 (44)		97 (43)		72 (46)		0.6
T2DM, *n* (%)	67 (17)		30 (13)		37 (24)		**0.01**
Fasting glucose, mg/dL	120 ± 47		114 ± 40		129 ± 55		1.0
Dyslipidaemia, *n* (%)	112 (29)		58 (26)		54 (35)		0.06
HDL, mg/dL	41 ± 17		41 ± 19		39 ± 15		0.3
Triglycerides, mg/dL	127 [100–167]		110 [86–161]		137 [111–173]		**0.001**
LDL, mg/dL	94 ± 45		92 ± 44		97 ± 46		0.7
Number of metabolic comorbidities^b^							
0, *n*%	90 (33)		51 (34)		39 (32)		0.1
1–2, *n*%	145 (54)		84 (56)		61 (50)		0.1
3–4, n%	36 (13)		14 (9)		22 (18)		**0.05**
Liver alterations							
Baseline ALT, IU/L	33 [19–53]		29 [17–47]		41 [22–73]		** < 0.001**
ALT > 2ULN, n (%)	46 (12)		19(8)		27(17)		**0.01**
Baseline AST, IU/L	16 [16–53]		16 [16–35]		39 [16–75]		** < 0.001**
AST > 2ULN, *n* (%)	56 (15)		22 (19)		34 (22)		**0.002**
ALT > 2ULN and/or AST > 2ULN, *n* (%)	692 (18)		27 (12)		42 (27)		** < 0.001**
Hepatic steatosis							
By HSI^c^	186 (70)		105 (56)		81 (43)		0.7
By US/CT^d^	82 (62)		41 (49)		42 (51)		0.3
FIB4^§^							
< 1.45	199 (54)		138 (69)		61 (31)		** < 0.001**
1.45–3.24	123 (34)		64 (52)		59 (48)		
> 3.25	45 (12)		16 (36)		29 (64)		
Markers of inflammation/thrombosis							
Basal ferritin mcg/L	721 [352–1261]		561 [276–1035]		1034 [576–1646]		** < 0.001**
> 1000 mcg/L, *n* (%)	141 (37)		61 (27)		81 (52)		** < 0.001**
Basal D-dimer, mcg/L	952 [550–1876]		869.5 [522–1680]		959 [587–1920]		0.1
> 500 mcg/L, *n* (%)	302 (79)		169 (75)		131 (84)		0.06
> 1000 mcg/L, *n* (%)	179 (47)		106 (47)		73 (47)		1.00
Basal IL-6	36.35 [12.7–82.4]		19.2 [7.7–41.0]		65 [22.1–125.5]		** < 0.001**
> 2ULN, *n* (%)	245 (64)		147 (65)		98 (63)		0.9
Basal CRP, mg/dL	5.8 [2.4–12.0]		4.1 [1.4–9.7]		9.3 [4.5–14.6]		** < 0.001**

*BMI* body mass index, *T2DM* type 2 diabetes, *HDL* high-density lipoprotein, *LDL* low-density lipoprotein, *AST* aspartate aminotransferase, *ALT* alanine aminotransferase, *GGT* gamma glutamyl transferase, *FIB4* fibrosis-4, *ULN* upper limit of normality, *CRP* C-reactive protein

^a^Severity of SARS-CoV-2 infection if necessity of CPAP/ICU and/or P/F < 200 and FR > 30

^b^BMI data available in 271 patients

^c^HSI data available in 266 patients

^d^US/CT scan data available in 133 patients

^§^FIB4 calculated in 367 patients

Bold values indicate that *p* is statistically significant

#### Hepatic disease

AST and ALT > 2 ULN were present in 12% and 15% of cases (Table 1).

Hepatic steatosis was diagnosed in 82 patients (62%), while FIB-4 > 3.25 was present in 45 (12%) and < 1.45 in 199 (54%) (Table 1). Because of small number of patients with FIB-4 > 3.25, in the analysis, we considered as variable FIB4 < 1.45.

## COVID-19 severity

Forty-one percent of our cohort presented a severe form of COVID-19 disease. Among them, 82% required non-invasive or mechanical ventilation at admission or during hospitalization, whereas 80% had a P/F < 200 at presentation (mean P/F 260 ± 105) (Supplementary Table 1).

### COVID-19 severity associates with inflammation, metabolic comorbidities, and liver disease

Ferritin > 1000 mcg/L, D-dimer > 500 mcg/L, and IL-6 > 2ULN were observed in 37%, 79%, and 64% of patients, respectively, with a significantly higher prevalence in patients with severe SARS-CoV-2 (Table 1).

Patients with severe COVID-19 presented a significantly higher prevalence of T2DM (24% vs 13%, p = 0.01) and a slightly higher prevalence of dyslipidaemia (35% vs 26%, *p* = 0.06) compared to those with a non-severe disease (Table 1; Supplementary Fig. 1). When considering coexisting metabolic alterations, the presence of ≥ 3 alterations was significantly higher compared to non-severe COVID-19 (18% vs 9%, p = 0.05).

Features of liver disease were significantly different in patients with and without severe COVID-19, in terms of increased transaminases levels on admission (< 0.001), and FIB-4 values (< 0.001). No difference in the presence of hepatic steatosis was found between severity groups (Table 1).

**Fig. 1:**
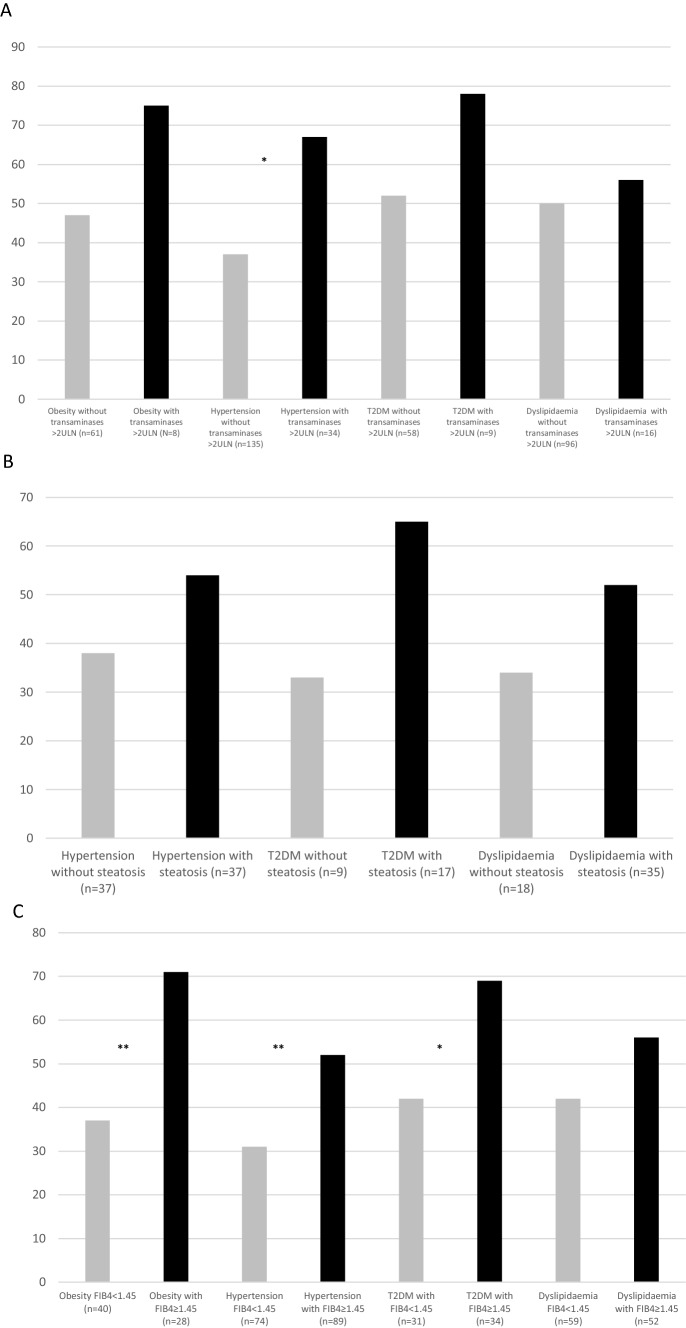
Prevalence of SARS-CoV-2 infection in patients with metabolic comorbidities stratified by liver disease. Bars represent prevalence of severe SARS-CoV-2 infection in patients with obesity, hypertension, type 2 diabetes (T2DM), and dyslipidaemia with (black) or without (grey) baseline transaminases > 2ULN (**A**), with (black) or without (grey) hepatic steatosis (**B**), with (grey) FIB-4 < 1.45 or ≥ 1.45 (black) (**C**)

Figure 1 reports the impact of any marker of liver disease severity on top of metabolic comorbidities. Specifically, the presence of transaminases > 2ULN was associated with severe COVID-19 only in patients with arterial hypertension (A). The impact of steatosis was negligible in any metabolic comorbidity group (B). Conversely, FIB-4 significantly mitigated the severity of COVID-19 in each group of metabolic comorbidities (C).

#### Impact of metabolic comorbidities adjusted for liver disease and markers of inflammation.

At multivariable analysis (Table 2), the burden of metabolic comorbidities was independently associated with a higher risk of severe COVID-19 and the higher the number of metabolic alterations, the higher the risk (for 1–2 metabolic alterations: OR 4.4, CI 95% 1.2–15.7; for 3–4 metabolic alterations: OR 5.4, CI 95% 1.1–29.6). Also, length of hospitalization (OR 1.06, CI 95% 1.02–1.09) resulted in an independent risk factor, whereas FIB-4 < 1.45 was protective (OR 0.3, CI 95% 0.1–0.8), losing this association when analyzed in the subset of patients who were aged > 65 years (patients with FIB5 < 1.45 and aged > 65 yrs *n *= 70) (data not shown). Basal transaminases > 2ULN was an independent risk factor for severe SARS-CoV-2 infection (OR 2.6, CI 95% 1.3–6.7) when added to the model instead of FIB-4 and hepatic steatosis; however, transaminases were not included in the final model because of collinearity. Association between metabolic alterations, FIB-4, and severity of COVID-19 was confirmed after adjustment for steroidal therapy, antiviral therapy, and low-weight heparin (data not shown).

**Table 2:** Multivariate analysis for severe SARS-CoV-2 infection (*n *= 156 patients). Metabolic comorbidities were introduced as burden of comorbidities by considering patients without any metabolic feature as a reference category of risk

	OR	95%-CI	*P*
Age	0.9	0.9–1.01	0.1
Sex	0.6	0.2–1.5	0.2
Comorbidities			**0.04**
0	ref	ref	
1–2	4.4	1.2–15.7	
3–4	5.4	1.1–29.6	
Hepatic steatosis	1.5	0.5–4.9	0.5
FIB4 < 1.45	0.3	0.1–0.8	**0.01**
Basal ferritin > 1000 mcg/dL	2.7	0.9–8.3	0.07
Length of hospitalisation	1.06	1.02–1.09	**0.006**

Patients with hematologic diseases and transaminases > 5ULN were excluded from the analysis (*n *= 10)

Bold values indicate that *p* is statistically significant

To differentiate the impact of any metabolic alteration, alone or in combination, on the severity of COVID-19 presentation, we performed the unadjusted and multivariate analysis, which was corrected for markers of liver disease (transaminases > 2ULN and FIB-4 < 1.45) and inflammation (Table 3).

**Table 3:** Association between metabolic comorbidities (either considered alone or in different combinations) and SARS-CoV-2 infection severity in unadjusted and multivariate models

	*n*	Unadjusted			Model 1 adjusted for basal transaminases > 2ULN			Model 2 adjusted for FIB4 < 1.45			Model 3 adjusted for FIB4 < 1.45 and basal ferritin > 1000 mcg/L		
		OR	CI 95%	*p*	OR	CI 95%	*p*	OR	CI 95%	p	OR	CI 95%	p
Single metabolic alterations													
T2DM	67	2.0	1.2–3.5	**0.009**	2.2	1.3–3.8	**0.004**	2.1	1.2–3.6	**0.01**	2.4	1.4–4.4	**0.003**
Dyslipidaemia	112	1.5	1–2.4	**0.06**	1.6	1.0–2.6	**0.003**	1.6	1.0–2.6	**0.04**	1.7	1.1–2.7	**0.03**
Hypertension	169	1.1	0.8–1.7	0.5	1.1	0.7–1.7	0.6	1.0	0.7–1.6	0.8	1.1	0.7–1.8	0.8
Obesity	69	1.6	0.8–2.9	0.1	1.4	0.8–2.6	0.2	1.5	0.9–2.7	0.1	1.6	0.9–3.0	0.1
Combination of two metabolic alterations													
T2DM + Dyslipidemia	32	3.0	1.4–6.5	**0.004**	3.4	1.6–7.5	**0.002**	3.2	1.5–7.1	**0.003**	3.5	1.5–7.8	**0.003**
T2DM + Hypertension	47	2.4	1.3–4.4	**0.006**	2.5	1.3–4.8	**0.004**	2.4	1.3–4.7	**0.007**	2.9	1.5–5.7	**0.002**
T2DM + Obesity	18	2.7	0.9–7.2	0.06	3.0	1.1–8.4	**0.03**	3.0	1.1–8.3	**0.04**	4.2	1.5–12.3	**0.008**
Obesity + hypertension	36	1.1	0.5–2.2	0.8	1.2	0.6–2.5	0.6	1.2	0.6–2.5	0.6	1.4	0.6–2.9	0.8
Obesity + Dyslipidaemia	26	1.7	0.8–4.0	0.2	2.1	0.9–4.8	0.08	2.0	0.9–4.7	0.09	2.3	0.9–5.8	0.06
Dyslipidaemia + hypertension	67	1.7	1.0–3.0	**0.03**	1.8	1.0–3.1	**0.03**	1.9	1.1–3.2	**0.03**	1.9	1.1–3.3	**0.03**
Combination of three metabolic alterations													
T2DM + dyslipidaemia + hypertension	27	3.1	1.4–7.2	**0.007**	3.6	1.6–8.4	**0.002**	3.6	1.5–8.7	**0.003**	4.0	1.6–9.7	**0.002**
T2DM + hypertension + obesity	12	3.9	1.0–14.7	**0.04**	4.4	1.2–16.9	**0.03**	4.0	1.1–15.6	**0.04**	5.2	1.3–20.9	**0.02**
Obesity + dyslipidaemia + hypertension	16	2.1	0.8–6.2	0.1	2.9	1.0–8.3	**0.04**	2.6	0.9–7.5	0.08	3.1	0.9–9.8	0.06
T2DM + dyslipidaemia + obesity	7	–	–	–	–	–	–	–	–	–	–	–	–
Combination of four metabolic alterations													
T2DM + dyslipidaemia + hypertension + obesity	5	–	–	–	–	–	–	–	–	–	–	–	–

For model including FIB4, patients with haematologic disease and/or basal ALT > 150 excluded (TD2M *n *= 66; dyslipidaemia *n *= 111, hypertension *n *= 163; obesity *n *= 68; T2DM + dyslipidaemia *n *= 32; T2DM + hypertension *n *= 46, T2DM + Obesity *n *= 18; Obesity + hypertension *n *= 36; Obesity + dyslipidaemia *n *= 26; dyslipidaemia + hypertension *n *= 66; T2DM + dyslipidaemia + hypertension *n *= 27; T2DM + hypertensio*n *+ obesity *n *= 12; obesity + dyslipidaemia + hypertension *n *= 16; T2DM + dyslipidaemia + obesity *n *= 7; T2DM + dyslipidaemia + hypertension + obesity *n *= 5)

Bold values indicate that *p* is statistically significant

Both T2DM and dyslipidaemia resulted in independent risk factors for severe SARS-CoV-2 disease in unadjusted and all multivariate models.

When considering the coexistence of two metabolic comorbidities, any combination including T2DM was associated with an increased risk of severe COVID-19 presentation in both unadjusted and all multivariate models, reaching the highest OR when combined with dyslipidaemia (Table 3. OR 3.4, CI 95% 1.6–7.5 model 1; OR 3.2, CI 95% 1.5–7.1 model 2; OR 3.4, CI 95% 1.5–7.8 model 3).

As for three metabolic comorbidities, almost all combinations of metabolic alterations conferred an increased risk of severe COVID-19, the highest represented by the association of T2DM with hypertension and obesity (OR 4.4, CI 95% 1.2–16.9 model 1; OR 4.0, CI 95% 1.1–15.6 model 2; OR 5.2, CI 95% 1.3–20.9 model 3).

For the combination of T2DM with dyslipidaemia and obesity, as well as coexistence of four metabolic alterations, statistical analysis could not be performed because of the low number of patients with this clinical condition in our series (*n *= 7 and *n *= 5, respectively).

### Impact of metabolic comorbidities and liver disease on SARS-CoV-2 mortality

In-hospital death was registered in 54 (14%) patients (Supplementary Table 1).

In patients who died compared to those who survived, a higher prevalence of hypertension (19% vs 10%, *p* = 0.02) and dyslipidaemia (23% vs 10%, *p* = 0.002), as well as of FIB-4 < 1.45 (64% vs 36%, *p* = 0.001) was found.

The burden of coexisting comorbidities was an independent risk factor for mortality (OR 4.6, CI 95% 1.0–21.6; *p* = 0.05), whereas an FIB-4 < 1.45 resulted in a protective factor (OR 0.4; CI 95% 0.1–0.9; *p* = 0.04) (Supplementary Table 2).

Median length of hospitalization was 14 days (min–max 2–82 days). No difference in the length of hospitalization was found according to the presence versus the absence of metabolic alterations, as well as hepatic steatosis. Conversely, as expected, patients with increased transaminases at admission presented longer hospitalization compared to those without it (increased ALT *p* = 0.002; AST < 0.001), possibly due to the expression of more severe disease requiring longer hospitalization. Similarly, as expected, patients with severe COVID infection had longer hospitalization compared to those without it. Finally, older patients had longer hospital stays compared to younger ones (data not shown).

## Discussion

The present study confirms the influence of metabolic alterations on COVID-19 severity and mortality, and highlights that the burden of metabolic comorbidities rather than a single metabolic feature has an independent role on the outcome of the infection. In addition, the FIB-4 score seemed to have a modulating effect on the unfavorable metabolic profile. This finding is of great interest, since, despite vaccination programs, COVID-19 infection remains today one of the main public health problems in patients at risk and identifying the key prognostic determinants of severity of the disease may help better focus health resources.

In spite of the established role of metabolic comorbidities during SARS-CoV-2 infection, to our knowledge, this is the first study reporting the impact of the burden of coexisting comorbidities on SARS-CoV-2 clinical course. In fact, the risk of severe SARS-CoV-2 reached more than a sevenfold increase in the presence of at least three metabolic comorbidities, even when the analysis was adjusted for liver disease and inflammation, thus hypothesizing a possible interplay of coexisting abnormalities able to amplify the damage during SARS-CoV-2 infection.

Among all metabolic alterations and in line with two recent meta-analysis by Kumar et al. [22] and Singh et al. [23], T2DM was the strongest risk factor for SARS-CoV-2 infection, either considered alone or combined. Conversely, dyslipidaemia was a mild risk factor for the severity of the respiratory infection, whereas arterial hypertension and obesity were not. Our results confirm observations from studies in Asians showing an increased risk linked to dyslipidaemia [24] and give a contribution to the still debated prognostic role of arterial hypertension on COVID-19 severity [25]. As for obesity, literature strongly supports its role in respect to SARS-CoV-2 severity and mortality [5, 26]; nevertheless, the lack of data about BMI in up to 30% of the series could partly explain this discrepancy.

In the present study, we also evaluated the role of hepatic disease on the severity of SARS-CoV-2 infection and its impact in the presence of metabolic comorbidities. In our series, liver fat seemed neither to influence the course of SARS-CoV-2 infection nor to modulate the effect of metabolic alterations. Indeed, although information on hepatic steatosis was available in only one-third of the cohort, the high prevalence of fatty liver in this subgroup of patients (60%) suggests that steatosis itself could be a risk factor for hospitalization for COVID-19, as supported by an American study including 6700 patients [27]. The lack of association between steatosis and severity of SARS-CoV-2 disease has been shown also by Mushtaq et al. [8] in a cohort of 589 Arabic patients, whereas other studies have reported opposite results in Asiatic populations and in subjects younger than 60 years [28, 29].

FIB-4 values, either considered as surrogate of hepatic fibrosis or simply as markers of liver damage, were significantly associated with COVID-19 disease severity. Indeed, having an FIB-4 < 1.45 resulted protective against severe SARS-CoV-2 infection. Our results mirror those reported by Targher et al. [10] in 310 Chinese COVID-19 patients with steatosis, showing that hepatic fibrosis assessed by FIB-4 at admission, conferred up to 6 times the risk of a severe form of respiratory disease. Similarly, recent evidence and a recent meta-analysis showed that high FIB-4 values were associated with a higher risk of mortality in patients with SARS-CoV-2 infection [30, 31]. It is known how chronic liver disease, especially if fibrosis coexists, is characterized by an increase in circulating inflammatory cytokines, which may worsen the known cytokine storm that is typical of COVID-19 disease, thereby fostering a more severe clinical deterioration [32, 33]. On the other hand, the inflammatory hallmark of COVID infection could promote liver damage, detected by increased transaminases, especially if fibrosis is present [34].

In addition to this evidence, we also showed that low FIB-4 score mitigated the negative effect of metabolic comorbidities on the severity of SARS-CoV-2 disease.

Finally, patients with increased transaminases had a threefold risk of severe COVID-19 independently of the burden of coexisting comorbidities and inflammation, though the rise in transaminases only slightly increased the risk in the presence of metabolic alterations, mainly in hypertensive patients. Nonetheless, the causality of this relationship could not be defined.

As for mortality from COVID-19, we reported an increased prevalence of dyslipidaemia and hypertension in patients who died compared to those who survived. Our results showed again that not only the single metabolic alteration but also the burden of coexisting comorbidities resulted as an independent prognostic factor for mortality, with a risk of approximately five times higher compared to those without metabolic alterations. As for the severity of the acute respiratory syndrome, normal FIB-4 values confirmed their protective role.

Our study has some limitations. First, the retrospective design prevents from drawing any causal relationship between metabolic alterations and the severity of SARS-CoV-2 infection, so a prospective analysis is warranted. However, our results are supported by many reports in the literature that have evidenced the role of metabolic alterations on COVID-19 clinical course. Second, our cohort includes subjects admitted to our ward from different clinical settings, therefore observed in different stages of COVID-19 disease, either at their first clinical presentation or after having already received more intensive care. Nonetheless, all biochemical and metabolic clinical data were collected at the time of their first examination regardless of the hospital setting. Third, data about hepatic steatosis was available in only a subset of patients, and diagnosed either with US or CT scan, which are not the gold standard to detect hepatic fat, especially of a mild entity. However, as reported previously, information about steatosis was retrieved by exams performed for other reasons during hospitalization and this is related to the retrospective design of the study. Nevertheless, US is routinely performed in clinical practice to detect hepatic steatosis, as suggested by the international guidelines, and we found a high percentage of patients with this condition, so that a bias in underestimating mild steatosis could have been overcome [19]. In addition, data about insulin-resistance, which is involved in the pathogenesis of hepatic steatosis, were not available. However, blood insulin determination was not routinely performed in COVID units. Finally, FIB-4 could not be considered a certain marker of hepatic fibrosis in our series because of a possible influence of hypertransaminasemia, usually described during COVID-19 infection [1]. However, in our analysis, this score was considered for a ruling-out strategy which prevented this bias. Along this line, the impact of liver alterations on top of metabolic comorbidities gave the same results by substituting FIB-4 with increased transaminases. Certainly, independently of the significance assumed by the FIB-4, this score could be applied as a useful tool to evaluate the risk of severe SARS-CoV-2.

## Conclusions

We showed in a cohort of Caucasian patients that collecting at admission simple information, such as metabolic comorbidities, as well as calculating an easy non-invasive fibrosis score such as the FIB-4, may help clinicians in identifying patients at risk of developing severe forms of SARS-CoV-2 infection, possibly requiring hospitalization.

In fact, we demonstrated that the number of metabolic alterations has a multiplicative effect on the severity of COVID-19 and applying the FIB-4 score, as a possible expression of fibrosis, may further stratify the risk of severe respiratory disease. Finally, the high prevalence of hepatic steatosis in our cohort raises questions on its role in predisposing to hospitalization due to SARS-CoV-2.

In conclusion, our results may help clinicians in managing patients with SARS-CoV-2 infection since the knowledge of predisposing/modulating factors is crucial given the continuously evolving scenario of this pandemic and the current health emergency despite vaccination programs.

## Supplementary Information

Below is the link to the electronic supplementary material.

Supplementary file1 (PDF 57 KB)

Supplementary file2 (DOCX 15 KB)

## Abbreviations

of abbreviations: SARS-CoV-2: Severe Acute Respiratory Syndrome Coronavirus-2
ULN: Upper limit of normality
FIB-4: Fibrosis-4
T2DM: Type 2 diabetes
ICU: Intensive Care Unit
NAFLD: Non-alcoholic fatty liver disease
RT-PCR: Real-time reverse-transcription polymerase chain reaction
BMI: Body mass index
Hb1Ac: Glycated hemoglobin
LDL: Low-density lipoprotein
HDL: High-density lipoprotein
AST: Aspartate aminotransferase
ALT: Alanine aminotransferase
GGT: Gamma-glutamyltransferase
HIS: Hepatic steatosis index
US: Ultrasound
CT: Computed tomography
P/F: PaO2/FiO2 ratio
RR: Respiratory rate
IL-6: Inteleukin-6
SD: Standard deviation
IQR: Interquartile range
OR: Odd ratio
