# Supplementary material for: Usefulness of fibrosis-4 (FIB-4) score and metabolic alterations in the prediction of SARS-CoV-2 severity
Source: Intern Emerg Med. 2022 Jun 26;17(6):1739–49. doi: 10.1007/s11739-022-03000-1 (PMC9244481; doi:10.1007/s11739-022-03000-1)
Supplement: Supplementary file 1 — Supplementary file1 (PDF 57 KB) [file 11739_2022_3000_MOESM1_ESM.pdf]

Supplementary figure 1.

Prevalence of metabolic comorbidities according to SARS-COV-2 infection severity (n=382). Bars represent prevalence of Non-severe SARS-COV2 infection (grey, n=226) and severe SARS-COV2 infection (black, n=156) in patients with obesity, hypertension, type 2 diabetes (T2DM) and dyslipidemia. Prevalence expressed as %. Significance for p<0.05(*) and p<0.1 (**)

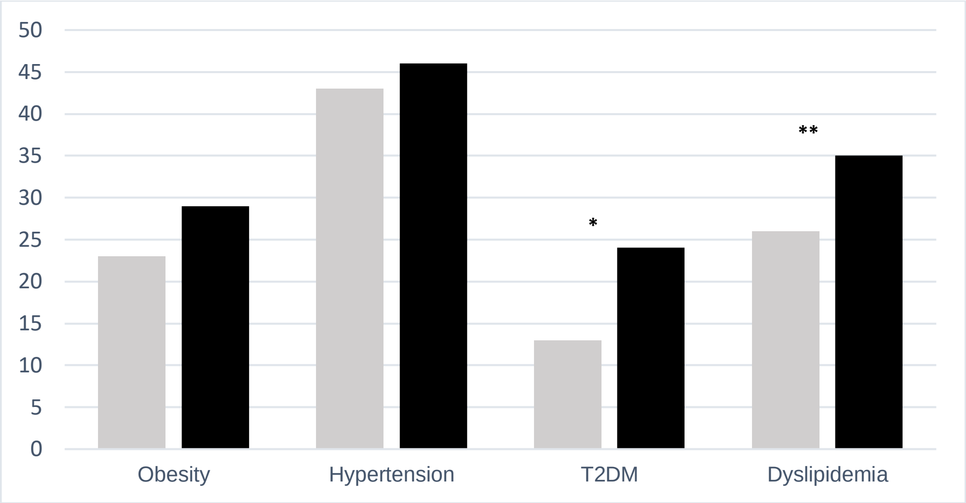

*data on obesity available in 271 patients (not severe= 149, severe=122)
ABBREVIATIONS: T2DM: type 2 diabetes mellitus
