# Supplementary material for: Usefulness of fibrosis-4 (FIB-4) score and metabolic alterations in the prediction of SARS-CoV-2 severity
Source: Intern Emerg Med. 2022 Jun 26;17(6):1739–49. doi: 10.1007/s11739-022-03000-1 (PMC9244481; doi:10.1007/s11739-022-03000-1)
Supplement: Supplementary file 2 — Supplementary file2 (DOCX 15 KB) [file 11739_2022_3000_MOESM2_ESM.docx]

**Supplementary Table 1.** Features of severity of SARS-COV2 infection in the whole cohort of patients (n=382)

| **Characteristics** | **N= 382** |
| --- | --- |
| Severe SARS-CoV-2 infection, n (%)  Of whom,  CPAP/ICU, n (%)  P/F < 200, n (%)  P/F, ratio  RR > 30, n (%)  P/F<200 and FR>30, n(%) | 156 (41)  128 (82)  125 (80)  260 ± 106  60 (38)  53 (14) |
| In-hospital mortality, n (%) | 54 (14) |

ABBREVIATIONs: CPAP: continuous positive airway pressure; ICU: intensive care unit.

**Supplementary Table 2.** Multivariate analysis of the association between metabolic comorbidities (either alone or combined) and mortality from SARS-COV2 infection (analysis adjusted for age and sex and for variables significantly associated with SARS-COV2 severity in multivariate analysis)

|  | **OR** | **CI 95** | **P** |
| --- | --- | --- | --- |
| Age, ys | 1.06 | 1.02-1.09 | **0.001** |
| Sex, male | 0.7 | 0.3-1.9 | 0.5 |
| Comorbidities |  |  | **0.05** |
| 0 | ref | ref |  |
| 1-2 | 4.6 | 1.0-21.6 |  |
| 3-4 | 1.4 | 0.2-10.4 |  |
| FIB4<1.45* | 0.4 | 0.1-0.9 | **0.04** |
| Basal ferritin >1000 mcg/dL | 0.8 | 0.3-2.1 | 0.7 |

*Multivariate analysis performed without age in order to avoid collinearity.

Patients with hematological diseases and transaminases >150 U/L were excluded from the analysis (n=10).

No association of transaminases even when FIB4 not added to the analysis
